# Cumulative Hydrocortisone Exposure and Early Brain Volumetrics in Very Low Birth Weight Infants: Associations with Neurodevelopmental Outcomes

**DOI:** 10.3390/biomedicines13112765

**Published:** 2025-11-12

**Authors:** Min Soo Kim, Moon-Yeon Oh, Emi Tomita, Soo-Ah Im, Young-Ah Youn, Sae Yun Kim

**Affiliations:** 1 Department of Pediatrics, Seoul St. Mary’s Hospital, College of Medicine, The Catholic University of Korea, 222 Banpo-daero, Seocho-gu, Seoul 06591, Republic of Korea; kms9057@gmail.com (M.S.K.); omygo0d@hanmail.net (M.-Y.O.); lea732@hanmail.net (Y.-A.Y.); 2 Artificial Intelligence Research Center, JLK Inc., Seoul 06141, Republic of Korea; emijong@gmail.com; 3 Department of Radiology, Seoul St. Mary’s Hospital, College of Medicine, The Catholic University of Korea, Seoul 06591, Republic of Korea; saim@catholic.ac.kr

**Keywords:** hydrocortisone, very low birth weight infants, brain development, magnetic resonance imaging, brain volume, neurodevelopmental outcomes

## Abstract

**Background/Objectives**: Systemic hydrocortisone (HCS) in very low birth weight (VLBW) infants is commonly used to treat early hypotension or prevent bronchopulmonary dysplasia. This study evaluated the associations between postnatal HCS exposure and neurodevelopment in VLBW infants by comparing regional brain volume at term-equivalent age (TEA) with neurodevelopmental outcomes in early infancy. **Methods**: This retrospective cohort study included VLBW infants admitted to a neonatal intensive care unit (NICU) between 2013 and 2019. The cumulative HCS dose during hospitalization was recorded, and regional brain volumes were analyzed using magnetic resonance imaging at TEA. Neurodevelopmental outcomes were assessed at a corrected age for prematurity of 18–24 months. **Results**: Among 146 infants, 57 were classified in the high HCS group (>90 mg/kg) and 89 in the low HCS group (≤90 mg/kg HCS). Bronchopulmonary dysplasia, periventricular leukomalacia, and sepsis were more frequent in the high HCS group. Ninety-five infants underwent magnetic resonance imaging, which revealed reduced brain volumes in the high HCS group. At follow-up, cerebral palsy (35.9% vs. 9.1%, *p* = 0.003), neurodevelopmental impairment (54.0% vs. 23.6%, *p* = 0.002), and head circumference <10th percentile (64.3% vs. 19.5%, *p* < 0.001) were more common in the high HCS group. After adjustment, HCS > 90 mg/kg remained independently associated with cerebral palsy (adjusted odds ratio [aOR] 5.44, *p* = 0.016) and reduced head circumference (aOR 4.45, *p* = 0.016). **Conclusions**: High cumulative HC exposure correlated with reduced brain volume at TEA and adverse neurodevelopmental outcomes at 24 months of age. Careful monitoring of dose and treatment duration is essential to balance therapeutic benefits against potential risks.

## 1. Introduction

Preterm birth remains one of the leading causes of neonatal morbidity and mortality worldwide. Advances in perinatal and neonatal intensive care have markedly improved the survival of very low birth weight (VLBW) infants, yet these fragile survivors remain at high risk of complications. Among these complications, respiratory insufficiency and circulatory instability are particularly frequent and often interrelated to challenges in the early postnatal period. The immature lungs of preterm infants are highly susceptible to inflammation and oxidative stress, while their cardiovascular system struggles to maintain adequate perfusion due to poor vascular tone, limited myocardial contractility, and an underdeveloped adrenal response [1,2,3]. Consequently, many preterm infants experience a complex interplay of systemic hypotension, prolonged ventilator dependence, and evolving bronchopulmonary dysplasia (BPD), which together contribute to adverse long-term neurodevelopmental outcomes.

Postnatal corticosteroids have been extensively used in neonatal intensive care units (NICUs) to enhance cardiovascular stability and mitigate pulmonary inflammation. They remain one of the most frequently administered pharmacologic agents in critically ill neonates, underscoring both their therapeutic value and the ongoing uncertainty regarding optimal indications, timing, and dosing [4]. According to a recent U.S. cohort study, 38% of extremely low birth weight infants were exposed to at least one corticosteroid [5].

These agents are employed for diverse indications—ranging from refractory hypotension to prevention or treatment of BPD—underscoring their central role in the management of extremely preterm infants. Systemic hypotension is a common and clinically significant complication following preterm birth. Reported incidence rates range from 20% to 45%, with prevalence increasing as gestational age decreases [6,7]. Although there is no universally accepted definition of “normal” blood pressure for preterm infants, Pejovic et al. demonstrated that the mean arterial pressure (MAP) tends to rise with GA [8]. The most commonly used definition of hypotension in preterm infants is a MAP (in mmHg) below the infant’s GA in weeks [9]. However, this criterion may not be appropriate for extremely preterm infants (GA < 25 weeks), whose cardiovascular adaptation is limited. In a cohort of infants born at 23–25 weeks, hypotension was defined as MAP ≤25 mmHg during the first 72 h of life [10,11]; after which MAP values persistently below 30 mmHg were considered abnormal. Prolonged hypotension during this early period has been linked to higher risks of intraventricular hemorrhage, periventricular leukomalacia (PVL), and mortality [12,13].

Despite its clinical significance, hypotension in neonates remains a controversial topic. Disagreements persist regarding its definition, indications for treatment, choice of therapies, and the potential risks and benefits associated with intervention [14,15]. Treatments vary widely among institutions and may include volume expansion, vasopressors, and corticosteroid supplementation [11,16,17].

Because pulmonary inflammation is a major risk factor for the development of BPD, postnatal corticosteroids are frequently administered to prevent or treat BPD in ventilator-dependent preterm infants. These drugs exert potent anti-inflammatory effects on the lungs, reducing airway inflammation and improving pulmonary function [1]. Several studies have shown that corticosteroids decrease the incidence of BPD and facilitate extubation in ventilator-dependent infants [18,19]. Watterberg et al. further demonstrated that low cortisol levels during the first postnatal week are associated with a higher risk of developing BPD, suggesting a potential benefit of early hydrocortisone (HCS) replacement therapy [20].

There is substantial variability in the literature regarding the type, timing, and dosage of systemic corticosteroids used [21,22]. Dexamethasone, a highly potent synthetic glucocorticoid, became widely used in the 1990s after reports of short-term respiratory benefits [23]. It has been shown to increase blood pressure in neonates unresponsive to fluid resuscitation and vasopressors, likely because of its cortisol-replacing effects in adrenal insufficiency [24,25]. However, concerns emerged as early or high-dose dexamethasone was associated with serious adverse effects, including neurodevelopmental impairment [26], intestinal perforation [27], and increased susceptibility to infection [28]. While dexamethasone remains an evidence-based therapy for reducing the risk for BPD in high-risk infants [29], its potential long-term neurotoxicity has prompted more cautious and restrictive use in current neonatal guidelines.

HCS, in contrast, has emerged as a more physiological alternative to dexamethasone, owing to its combined glucocorticoid and mineralocorticoid activity that may more closely mimic endogenous cortisol. It is widely used to treat vasopressor-resistant hypotension and to prevent BPD, due to its comparatively favorable safety profile. In previous studies, HCS has demonstrated efficacy in increasing blood pressure, particularly in cases of vasopressor-resistant hypotension, and is associated with relatively few complications [30]. However, it is not without risk; prolonged or high cumulative exposure has been linked to impaired brain growth, altered white matter development, and later neurobehavioral deficits [31]. However, pharmacokinetic data to guide optimal dosing in this vulnerable population is insufficient. Consequently, various “stress doses” are used in clinical practice, which may lead to excessive HCS exposure, particularly in extremely preterm infants [32]. Moreover, the relation between cumulative HCS dose and long-term neurodevelopmental outcomes is not clearly defined, highlighting the need to establish a dose threshold that could inform safer and more individualized treatment strategies.

Although HCS use has increased globally in NICUs, most studies have focused on short-term outcomes—such as respiratory status, hemodynamic stabilization, or survival—while its effects on the developing brain remain insufficiently explored [31,33,34,35,36,37,38,39]. Evidence regarding the optimal timing, cumulative dose, and long-term neurological safety of HCS is still inconsistent. Only a limited number of studies have evaluated neuroimaging outcomes, and most used cranial ultrasound or conventional qualitative magnetic resonance image (MRI) rather than quantitative techniques [40]. With the advent of volumetric MRI, regional brain volumes vulnerable to glucocorticoid exposure can now be measured more precisely [39,41]. However, data linking cumulative HCS exposure to brain volumetrics at TEA and subsequent neurodevelopment remain scarce. Identifying the optimal HCS dose is essential to balance its therapeutic benefits against potential neurodevelopmental risks in VLBW infants.

The primary objective of the present study was to assess the independent association between high cumulative HCS exposure and neurodevelopmental outcomes at 2 years of corrected age for prematurity (CA) in VLBW infants, while adjusting for potential confounders. The secondary objective was to compare brain volume at TEA using three-dimensional MRI between infants with differing cumulative postnatal HCS exposures.

## 2. Materials and Methods

### 2.1. Study Design and Subject Selection

**Figure 1:**
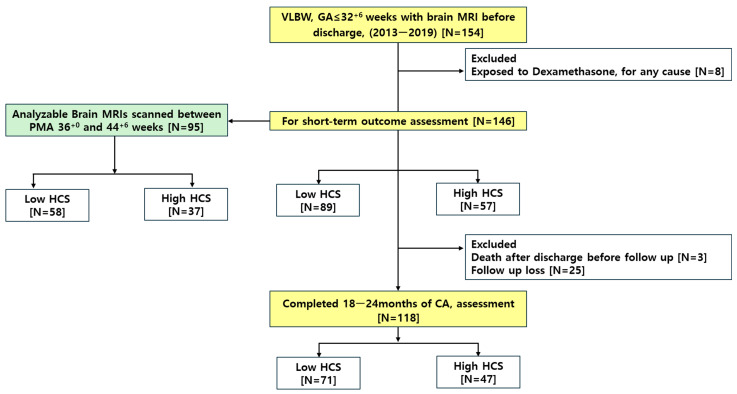
Selection flow of the study population. Abbreviations: CA, corrected age for prematurity; HCS, hydrocortisone; GA, gestational age; MRI, magnetic resonance imaging; PMA, postmenstrual age; VLBW, very low birth weight.

This was a retrospective cohort study. VLBW infants with a GA of ≤ 32^+6^ weeks who were born between 2013 and 2019 and admitted to the neonatal intensive care unit (NICU) of Seoul St. Mary’s Hospital were eligible. Infants exposed to dexamethasone for any reason were excluded from this study. Additionally, brain MRI scans acquired between 36^+0^ to 44^+6^ weeks postmenstrual age (PMA) were subjected to volumetric analysis. Moreover, those who survived until NICU discharge and were followed up 18–24 months of CA were selected for long-term outcome evaluation (Figure 1).

### 2.2. Administration of HCS in NICU: Hemodynamic Support and BPD Prevention/Treatment

For hypotensive newborns with poor perfusion or decreased urine output, intravenous HCS is one of the primary therapeutic options, provided there are no signs of infection. The standard initial dose was 1 mg/kg administered every 8 h, and the duration was adjusted based on the therapeutic response. After circulatory stabilization, the dose was reduced to 1 mg/kg every 12 h for 3–4 d, tapered to 0.5 mg/kg every 12 h for 7 d, and finally to 0.5 mg/kg once daily for 3 d. If the infant’s condition worsened after the initial dose, vasopressors were initiated, and the dose increased to 2 mg/kg every 6 h. In cases of further deterioration despite high-dose therapy, a single dose of 5 mg/kg was considered, in accordance with recommendations for adrenal crisis in neonates with congenital adrenal hyperplasia [42]. In addition, HCS was administered to infants at high risk of developing BPD, particularly those older than 14 d who had difficulty weaning from mechanical ventilation. An initial dose of 1 mg/kg was administered every 8 h, with subsequent dose adjustments made after 3 d based on the ventilator status.

### 2.3. Data Collection and Definitions

Maternal characteristics included age, hypertensive disorder of pregnancy (HDP) [43], mode of delivery, histological chorioamnionitis, and antenatal steroid use. Histological chorioamnionitis (HCA) was defined according to the criteria described by Yoon et al. [44]. The presence or absence of HCA was recorded as a categorical variable. Administration of antenatal corticosteroids (ACS) was defined as the mother receiving more than one dose of corticosteroids at any time before delivery. Neonatal variables included GA at birth, birth weight, 5 min Apgar score, sex, and small for gestational age (SGA) status. SGA was defined as a birth weight < 10th percentile for GA and sex [45]. Neonatal outcomes during NICU admission included respiratory distress syndrome, patent ductus arteriosus (PDA), moderate to severe BPD [1], necrotizing enterocolitis ≥ stage II [46], intraventricular hemorrhage exceeding grade 2 [47], PVL, retinopathy of prematurity [48], culture-proven sepsis, duration of mechanical ventilation, duration of parenteral nutrition, and length of NICU stay.

For long-term evaluation, CA were calculated by subtracting the number of weeks born before 40 weeks of gestation from the chronological age [49]. All infants were scheduled for a follow-up at 18–24 months of CA for a comprehensive assessment of growth and neurodevelopment. Neurodevelopmental assessments were conducted using the Bayley Scales of Infant and Toddler Development, Third Edition (BSID-III). Motor, language, and cognitive impairments were defined as scores < 85 in the respective domains. Cerebral palsy (CP) was defined as a Gross Motor Function Classification System (GMFCS) score ≥ II [50]. Neurodevelopmental impairment (NDI) was defined as the presence of CP with GMFCS ≥ II or impairment in any of the BSID-III domains.

### 2.4. MRI Acquisition and 3D Volumetric Assessment with Semi-Automatic Segmentation

**Figure 2:**
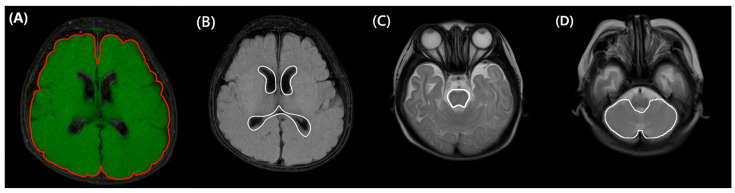
Regional structure marking: Intracranial (**A**) and ventricle (**B**) marking was drawn on an axial FLAIR T2 FAT sequence. Brain stem (**C**) and cerebellar (**D**) marking was drawn on a T2-Weighted TSE sequence. FLAIR, Fluid-Attenuated Inversion Recovery.

**Figure 3:**
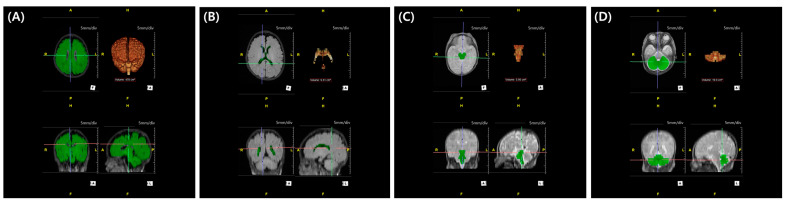
Regional volume calculated through 3D workstation: 3D-reconstructed intracranial volume (**A**), 3D-reconstructed ventricle volume (**B**), 3D-reconstructed brain stem volume (**C**), and 3D-reconstructed cerebellar volume (**D**).

In our center, brain MRI is routinely performed in very preterm or VLBW infants to screen for white matter injuries. According to our protocol, MRI is recommended at TEA. However, if the infant is medically unstable, the scan is delayed. Conversely, if earlier imaging is clinically indicated, MRI may be performed prior to TEA. Minimizing the confounding effect of brain growth, we selected brain MRI scanned between the previously mentioned window period: obtained between 36^+0^ to 44^+6^ weeks of PMA. Axial fluid-attenuated-inversion-recovery and T2 MRI images (Figure 2) were annotated by a technician (TE) using a 3D workstation (Aquarius iNtuition, TeraRecon Inc., Durham, NC, USA) program. The results were reviewed and confirmed by a pediatric radiologist (ISA). The evaluators were blinded to the clinical outcomes. Based on these annotations, quantitative assessment (total brain volume, cerebral volume, cerebellar volume, brainstem volume, and ventricular volume) was performed. The segmented structures were reconstructed in 3D to confirm shape accuracy; an example is shown in Figure 3.

### 2.5. Statistical Analysis

To determine the optimal cutoff value for the cumulative HCS dose for predicting CP, a receiver operating characteristic (ROC) curve analysis was performed. The area under the curve (AUC) was highest when the cutoff was set at 90 mg/kg. Accordingly, infants who received a cumulative HCS dose > 90 mg/kg before NICU discharge were classified into the high HCS group, whereas those who received ≤90 mg/kg were classified into the low HCS group.

Maternal and infant characteristics, as well as neonatal outcomes, were compared between the high HCS and low HCS groups using the Pearson chi-square test or Fisher’s exact test for categorical variables and either Student’s *t*-test or the Mann–Whitney U test for continuous variables, such as brain volumes. Logistic regression analyses were performed to assess the association between the cumulative HCS dose and long-term neurodevelopmental outcomes. The variance inflation factors were calculated to evaluate multicollinearity among variables in all multiple logistic regression models. All variables found to be statistically significant in the univariate models were included as potential confounders. Categorical variables are expressed as frequencies and percentages, and continuous variables are presented as means and standard deviations. Statistical analyses were conducted using R software package version 4.3.0 (R Foundation for Statistical Computing, Vienna, Austria) with a significance level of 0.05.

### 2.6. Ethical Statement

The study protocol was reviewed and approved by the Human Research Ethics Board of Seoul St. Mary’s Hospital (IRB no. KC23RASI0057, approved on 3 February 2023, and KC24RISI0775, approved on 3 December 2024). Informed consent from the parents of the study participants was waived due to the retrospective design of the study.

## 3. Results

A total of 154 VLBW infants with a GA of ≤32^+6^ weeks were included. Eight infants were excluded because of exposure to dexamethasone for any indication, leaving 146 infants for short-term outcome analysis. Among the infants, 23 were not exposed to HCS during their NICU admission. Of the remaining infants, 49 received HCS for hypotension, 23 were treated with HCS for the prevention or treatment of BPD, and 51 received HCS for both hypotension and BPD (Table A1). Additionally, we selected 95 MRI scans performed at TEA, between a PMA of 36^+0^ and 44^+6^ weeks to reduce age-dependent brain growth effect. For long-term developmental assessment, 118 infants with available data were included in the analysis of neurodevelopmental outcomes at 18–24 months CA (Figure 1).

### 3.1. Clinical Characteristics of the Study Population

The clinical characteristics of the study population, stratified by the cumulative HCS dose, are summarized in Table 1. Infants in the high HCS group had significantly lower GA (27.2 ± 2.0 weeks vs. 28.6 ± 2.1 weeks, *p* < 0.001) and birth weight (917.7 ± 248.9 g vs. 1133.7 ± 220.9 g, *p* < 0.001) than those in the low HCS group. Additionally, the 5 min Apgar score was lower in the high HCS group (5.1 ± 2.0 vs. 5.9 ± 1.7, *p* = 0.011). Significant differences were also observed in short-term neonatal outcomes, including higher incidence of moderate to severe BPD (100.0% vs. 69.7%, *p* < 0.001), treated PDA (49.1% vs. 28.1%, *p* = 0.016), PVL (49.1% vs. 28.1%, *p* = 0.016), and sepsis (56.1% vs. 32.6%, *p* = 0.008) in the high and low HCS group, respectively. The high HCS group also had significantly longer duration of mechanical ventilator (62.9 ± 38.8 days vs. 20.4 ± 27.5 d, *p* < 0.001) and hospital length of stay (112.3 ± 52.0 vs. 72.0 ± 32.4 d, *p* < 0.001).

### 3.2. Association Between Cumulative Hydrocortisone Dose and Brain Volume

**Figure 4:**
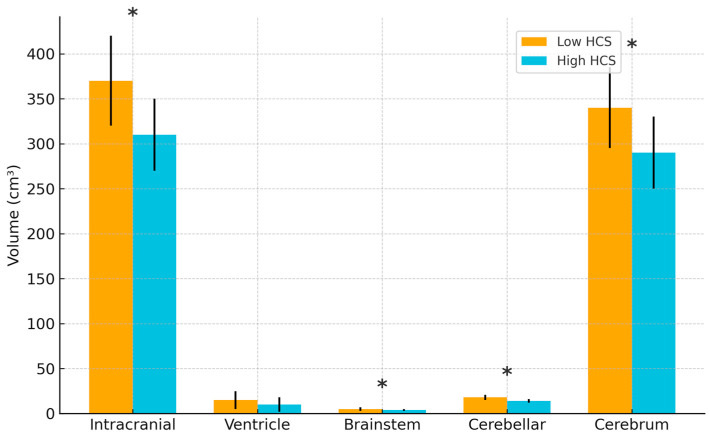
Brain volume according to the cumulative HCS dose. Comparison of brain volumes between low and high cumulative hydrocortisone (HCS) dose groups. Bars represent mean ± SD; * indicates *p* < 0.05. Abbreviations: HCS, hydrocortisone.

Figure 4 demonstrates significant differences in brain volumes depending on the cumulative HCS dose (Figure 4 and Table A2). Infants in the high HCS group exhibited reduced intracranial volume (309.7 ± 57.5 cm^3^ vs. 371.2 ± 66.1 cm^3^, *p* < 0.001), cerebellar volume (13.8 ± 5.8 cm^3^ vs. 19.6 ± 5.9 cm^3^, *p* < 0.001), and cerebral volume (292.5 ± 52.5 cm^3^ vs. 346.4 ± 61.8 cm^3^, *p* < 0.001) than those in the low HCS group. Brainstem volume was also significantly lower in the high HCS group (4.4 ± 0.8 cm^3^ vs. 5.3 ± 0.8 cm^3^, *p* < 0.001).

### 3.3. Association Between Cumulative Hydrocortisone Dose and Long-Term Outcome

**Figure 5:**
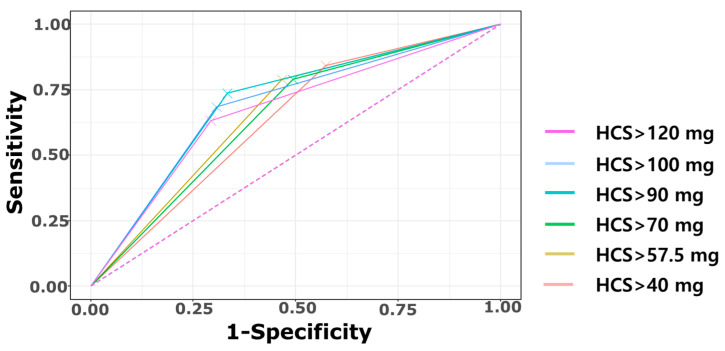
ROC curves for cerebral palsy prediction across cumulative hydrocortisone dose thresholds: ROC curves are shown for dose cutoffs of >40, >57.5, >70, >90, >100, and >120 mg/kg. The corresponding AUCs were 0.630, 0.661, 0.648, 0.702, 0.689, and 0.669, respectively. The >90 mg/kg cutoff was selected as the optimal threshold using the “largest-AUC” criterion (i.e., the cutoff yielding the maximum AUC among candidates). The dashed line represents the reference line (AUC = 0.5). Abbreviations: AUC, area under the curve; HCS, hydrocortisone; ROC, receiver operating characteristics.

Figure 4 presents the ROC curve analysis for discriminating CP according to cumulative HCS dose (mg/kg) cutoffs. Among the tested thresholds, 90 mg/kg yielded achieved the highest AUC (0.702) and Youden Index (0.404) and was therefore selected as the optimal cutoff using the largest-AUC criterion (Figure 5; Table A3). This threshold was subsequently used to examine the association between HCS dose and short-term outcomes as well as long-term morbidities.

The high HCS group showed a significantly higher incidence of CP (35.9% vs. 9.1%, *p* = 0.003) and NDI (54.0% vs. 23.6%, *p* = 0.002). Language impairment was notably more frequent in the high HCS group (59.5% vs. 33.3%, *p* = 0.017). Infants in the high HCS group had a higher prevalence of head circumference < 10th percentile (64.3% vs. 19.5%, *p* < 0.001) and height < 10th percentile (42.1% vs. 18.5%, *p* = 0.025). Although body weight at follow-up < 10th percentile was more common in the high HCS group (43.6% vs. 24.1%), this difference did not reach statistical significance (*p* = 0.073) (Table 2).

All significant factors identified in the univariate analysis were included in the multivariate logistic regression model as confounding factors (Table A4). After adjusting for confounders, the cumulative HCS dose > 90 mg/kg was significantly associated with CP (adjusted odds ratio [aOR] 5.44, 95% confidence interval [CI] 1.45–23.96, *p* = 0.016) and head circumference < 10th percentile (aOR 4.45, 95% CI 1.34–15.71, *p* = 0.016). Although NDI was more prevalent in the high HCS group, the association did not reach statistical significance (aOR 2.58, 95% CI 0.94–7.42, *p* = 0.071) (Table 3).

## 4. Discussion

To our knowledge, this is the first study to concurrently evaluate neonatal brain volume at TEA and long-term neurodevelopmental outcomes in relation to cumulative HCS exposure during the neonatal period. This study makes a significant contribution to the literature, by demonstrating that high cumulative HCS exposure in VLBW infants is associated with reduced brain volume at TEA and adverse neurodevelopmental outcomes during early infancy.

In preterm infants, HCS may offer neuroprotective benefits by reducing inflammation, stabilizing hemodynamics, and preserving cerebral perfusion [33,51,52,53], effects that can be particularly valuable for those with adrenal immaturity and refractory hypotension [54,55,56]. Early administration of replacement doses may help mitigate hemodynamic instability, pre-discharge mortality, and BPD. However, exogenous glucocorticoids can inhibit neuronal proliferation, particularly in the hippocampus and cerebral cortex [57], and MRI studies have linked higher cumulative HCS exposure to smaller total brain volumes at TEA [39]. Given that the third trimester is a critical period for rapid brain growth and maturation [58], preterm infants are especially vulnerable to insults during this critically developmental window [59]. Additional risks include spontaneous intestinal perforation (when used in combination with ibuprofen), late-onset sepsis, hyperglycemia, and hypertension. These opposing effects underscore the importance of individualized treatment decisions, with careful attention to timing, dosage, and duration, particularly because the optimal cumulative dose remains undefined [31].

In our study, infants in the high HCS group had significantly earlier GA, lower birth weight, and lower 5 min Apgar scores than those in the low HCS group. These findings are consistent with those of previous reports indicating that extremely preterm or critically ill neonates often require stress dose HCS [17,54,55]. These results highlight that HCS is often indispensable for managing extremely premature infants, particularly those with severe clinical instability. Consequently, cumulative HCS doses may be inevitably higher in such cases. Nevertheless, after adjusting for these possible confounders, the high HCS group continued to show poorer neurodevelopmental outcomes. These findings indicate that a high cumulative HCS dose linked to long-term neurodevelopmental harm may still exist, highlighting the need for the careful individualization of treatment timing, dosage, and duration.

Although HCS is generally regarded as neurodevelopmentally safer than dexamethasone [33], several studies have reported an association between HCS exposure and adverse outcomes. Patra et al. observed that HCS initiated after 7 days of life was associated with poorer fine motor outcomes at 8 months, as well as negative correlations with both language and motor development in early infancy [39]. They also reported a higher prevalence of microcephaly at 2 years in infants exposed to HCS, which is consistent with our results [60]. However, the long-term neurodevelopmental effects of early systemic HCS remain controversial. Some studies have shown no increase in the risk of CP with early low-dose HCS administration [61]. Similarly, Halbmeijer et al. reported that early HCS administration for BPD prevention did not increase the composite outcome of death or neurodevelopmental impairment at 2 years in ventilated very preterm infants [37].

In our cohort, the mean BSID-III scores in the low and high HCS groups, respectively, were 91.3 ± 15.7 versus 83.9 ± 17.2 for the cognitive domain, 89.5 ± 17.0 versus 79.5 ± 15.9 for language, and 84.4 ± 19.6 versus 73.7 ± 17.7 for the motor domain. These scores were lower than those reported for the term-born reference group by C. E. Green et al. (97.5 ± 11.2, 97.9 ± 16.0, and 98.2 ± 10.9 for the cognitive, language, and motor domains, respectively) [62]. Although both low and high HCS groups scored below term-born norms across cognitive, language, and motor domains at 24 months CA, deficits were greatest in the high-dose group. Low-dose exposure offered no clear neurodevelopmental disadvantage, whereas higher cumulative doses were associated with more pronounced impairment, raising concerns about the safety of high-dose use in this vulnerable population.

The timing and cumulative dose of HCS appear to be critical determinants of long-term neurodevelopmental outcomes. Meta-analyses of late treatment trials have shown a lower, yet still elevated, risk of adverse neurodevelopment, with a relative risk of 1.31 (95% CI, 0.85–2.01) and a number needed to harm of 20 [63]. By contrast, physiological or replacement dosing for adrenal insufficiency in critically ill neonates appears to be less harmful and may even provide benefits by stabilizing hemodynamics and reducing inflammatory injury. These findings suggest that HCS may carry risks under certain circumstances, such as a high cumulative dose. Taniguchi et al. reported that higher cumulative doses of HCS were associated with lower developmental and intelligence quotient scores at 6 years of age in extremely low birth weight infants [64]. However, to date, no consensus exists regarding a cumulative HCS threshold that predicts adverse outcomes.

In our cohort, the cumulative HCS dose showed an acceptable predictive ability for CP (AUC 0.702; sensitivity 73.7%; specificity 66.7%;) with the 90 mg/kg threshold (Figure 3). Previously, Puia-Dumitrescu et al. found that a mean cumulative dose of 3.1 mg/kg was associated with significantly poor motor and language development in early infancy [5]. When glucocorticoid potency was considered, the 90 mg/kg HCS threshold identified in our study was approximately equivalent to 3.6 mg/kg dexamethasone. Taken together, it is still considered a potentially safer alternative to dexamethasone. But, like dexamethasone, high cumulative HCS may also be associated with adverse neurodevelopmental outcomes once cumulative exposure exceeds a certain threshold. Considering the shared glucocorticoid effects of HCS and dexamethasone, it is reasonable to assume that high cumulative doses of either agent may contribute to brain toxicity through similar mechanisms.

Importantly, our study extends this body of evidence by simultaneously evaluating both neonatal brain volume at TEA and long-term neurodevelopmental outcomes, thereby providing a more comprehensive assessment of the potential risks associated with cumulative HCS exposure.

Several recent studies have reported a negative association between cumulative HCS exposure and total brain volume at TEA, including reductions in cortical gray matter and cerebellar volumes; Knickmeyer et al. measured mean total brain volume at 2–4 postnatal weeks in infants born after 34 weeks of gestation and reported a volume of 425.4 ± 48.1 cm^3^ [65]. Hüppi et al. assessed brain volumes in preterm and term infants scanned between 29 and 41 weeks’ PMA, with TEA brain volume approximating 400 cm^3^ [66]. These values align with the general estimate that the neonatal brain is approximately one-fourth to one-third the size of the adult brain [67]. In our study, infants in the low HCS group had a mean brain volume of 371.2 ± 66.1 cm^3^ at TEA, closely matching term volumes reported by Knickmeyer et al., suggesting that low-dose HCS exposure may not adversely affect brain growth.

The results of our study are consistent with previous research, suggesting that higher cumulative doses of HCS may adversely affect brain development. These phenomena might be linked with the neurotoxicity of glucocorticoids. Glucocorticoids may exert neurotoxic effects by exacerbating neuronal and astroglial injury in the developing brain. Cheong et al. called particular attention to the hippocampus, wherein they noted the suppression of neurogenesis, myelination, and cell proliferation, as well as selective vulnerability of the neonatal cerebellum owing to the high density of glucocorticoid receptors in the external granular layer [68]. In Cheong’s cohort, only the effect of dexamethasone was investigated. Considering the shared glucocorticoid effects of HCS and dexamethasone, it is reasonable to assume that high cumulative doses of either agent may contribute to brain toxicity via similar mechanisms.

There are several limitations to our study. First, due to its retrospective design, a definite causal relation between HCS exposure and long-term adverse neurodevelopmental outcomes cannot be established. Further randomized controlled trials or large-scale prospective studies are needed to confirm these findings. Second, regarding MRI volumetric analysis, the timing of brain MRI acquisition varied among participants, with a window ranging from 36^+0^ to 44^+6^ weeks of PMA. This wide time range may have introduced variability. Third, the effects and potential harm caused by corticosteroids may vary depending on the timing of their administration. Earlier exposure may have a more detrimental impact on the developing brain. Our analysis did not account for HCS administration during this critical period. Lastly, although our analysis demonstrated an association between cumulative HCS dose and TEA brain volume, it is possible that infants with poorer baseline clinical status had impaired brain growth regardless of HCS exposure. The lack of further adjustment for baseline severity of illness or risk stratification in the brain volume analysis is another limitation of our study, highlighting the need for future multivariate analyses that account for these potential confounders.

## 5. Conclusions

In conclusion, our study reinforces the idea that the use of HCS in preterm infants should be approached with caution. A higher cumulative dose of HCS for VLBW infants during the neonatal period is associated with decreased brain volume at TEA and impaired developmental outcomes in early infancy. While lower cumulative doses may not be associated with measurable neurodevelopmental harm, higher exposures appear to increase the risk of impairment, independent of other perinatal risk factors. Given the potential life-saving benefits and long-term adverse effects, treatment decisions should prioritize the lowest effective dose, individualized timing, and close follow-up of neurodevelopmental outcomes. Future research should aim to refine dosing thresholds and identify subgroups most likely to benefit from therapy while minimizing risk.

## Figures and Tables

**Table 1:** Clinical characteristics of the study population depending on the cumulative dose of hydrocortisone.

Characteristics	Low HCS [N = 89]	High HCS [N = 57]	*p*
Maternal Characteristics			
Maternal age, years	33.5 ± 3.9	33.2 ± 4.7	0.705
Maternal HDP	10 (11.2%)	8 (14.0%)	0.807
Delivery mode, C/S	73 (82.0%)	47 (82.5%)	>0.999
HCA	33 (37.1%)	21 (36.8%)	>0.999
Antenatal steroid	63 (75.0%)	40 (76.9%)	0.961
Infantile Characteristics			
Gestational age, weeks	28.6 ± 2.1	27.2 ± 2.0	<0.001
Birth weight, grams	1133.7 ± 220.9	917.7 ± 248.9	<0.001
5 min Apgar Score	5.9 ± 1.7	5.1 ± 2.0	0.011
Male	42 (47.2%)	31 (54.4%)	0.497
Small for gestational age	9 (10.1%)	10 (17.5%)	0.294
Short-term Neonatal Outcomes			
Respiratory distress syndrome	83 (93.3%)	55 (96.5%)	0.642
Treated patent ductus arteriosus	25 (28.1%)	28 (49.1%)	0.016
Moderate to severe BPD	62 (69.7%)	57 (100.0%)	<0.001
Necrotizing enterocolitis	7 (7.9%)	6 (10.5%)	0.800
Periventricular leukomalacia	25 (28.1%)	28 (49.1%)	0.016
Intraventricular hemorrhage ≥ grade 2	65 (73.0%)	45 (78.9%)	0.541
Treated retinopathy of prematurity	10 (11.2%)	5 (8.8%)	0.842
Culture proven sepsis	29 (32.6%)	32 (56.1%)	0.008
Duration of ventilator, d	20.4 ± 27.5	62.9 ± 38.8	<0.001
Duration of PN, d	49.4 ± 44.3	39.6 ± 28.8	0.104
Length of stay, d	72.0 ± 32.4	112.3 ± 52.0	<0.001

Values are expressed as mean ± SD or number (percentage). Abbreviations: HCS, hydrocortisone; C/S, cesarian section; HCA, histological chorioamnionitis; HDP, hypertensive disorder of pregnancy; BPD, bronchopulmonary dysplasia; PN, parenteral nutrition.

**Table 2:** Long-term outcomes depending on the cumulative dose of hydrocortisone.

		Low HCS	High HCS	*p*
Neurodevelopmental outcome	Cerebral palsy	5/55 (9.1%)	14/39 (35.9%)	0.003
	Motor impairment	24/57 (42.1%)	25/42 (59.5%)	0.131
	Language impairment	19/57 (33.3%)	25/42 (59.5%)	0.017
	Cognitive impairment	17/57 (29.8%)	17/42 (40.5%)	0.374
	NDI	13/55 (23.6%)	34/63 (54.0%)	0.002
Growth outcome	FU Body weight < 10th	14/58 (24.1%)	17/39 (43.6%)	0.073
	FU Height < 10th	10/54 (18.5%)	16/38 (42.1%)	0.025
	FU Head circumference < 10th	8/41 (19.5%)	18/28 (64.3%)	<0.001

Values are expressed as mean ± SD or number (percentage). Abbreviations: FU, follow-up; HCS, hydrocortisone; NDI, neurodevelopmental impairment.

**Table 3:** Multiple logistic regression analysis model between cumulative hydrocortisone dose and long-term outcomes.

	Low HCS [N = 71]	High HCS [N = 47]	aOR	95% CI	*p*
CP^a^	5/55(9.1%)	14/39(35.9%)	5.44	1.45–23.96	0.016
NDI^b^	13/55(23.6%)	34/63(54.0%)	2.58	0.94–7.42	0.071
HC < 10%^c^	8/41(19.5%)	18/28(64.3%)	4.45	1.34–15.71	0.016

aORs were adjusted for variables significantly associated with neonatal outcomes in the univariate analysis. CP is defined as a GMFCS level ≥ II. NDI is defined as the composite of any Bayley-III subdomain score < 85 or CP. A total of 94 infants completed the evaluation for CP^a^, 118 infants completed the evaluation for NDI^b^, and 69 infants completed head circumference measurement^c^ at 18–24 months of corrected age. Abbreviations: aOR, adjusted odds ratio; Bayley-III, Bayley Scales of Infant and Toddler Development–III; CI, confidence interval; CP, cerebral palsy; GMFCS, Gross Motor Function Classification System; HC, head circumference; HCS, hydrocortisone; NDI, neurodevelopmental impairment.

## Data Availability

The datasets analyzed in this study are not publicly available. The information contained in the data must be protected as confidential and will only become available to those individuals who have obtained permission from the data review board and IRB of Seoul St. Mary’s Hospital to access and use the data for permitted research activities. The original contributions presented in this study are included in the article materials. Further inquiries can be directed to the corresponding author.

## Appendix A

**Table A1:** Characteristics of HCS treatment.

Brain Volume	Without HCS [N = 23]	HCS for Low BP [N = 49]	Both Low BP and BPD [N = 51]	HCS for BPD [N = 23]
Cumulative HCS dose, mg/kg	NA	78.1 ± 58.9	168.9 ± 123.1	48.7 ± 54.5
Duration of HCS therapy, days	NA	18.0 ± 13.6	46.9 ± 33.0	11.7 ± 12.5
PND of HCS initiation, days	NA	3.9 ± 6.1	3.7 ± 7.4	18.0 ± 16.1
PMA at HCS initiation, weeks	NA	28.8 ± 2.4	27.7 ± 1.8	31.3 ± 2.7

Values are expressed as mean ± SD. Abbreviations: BPD, bronchopulmonary dysplasia; BP, blood pressure; HCS, hydrocortisone; PND, postnatal day; PMA, postmenstrual age.

**Table A2:** Brain volume depending on the cumulative dose of hydrocortisone.

Brain Volume	Low HCS [N = 58]	High HCS [N = 37]	*p*
Intracranial volume, cm^3^	371.2 ± 66.1	309.7 ± 57.5	<0.001
Ventricle volume, cm^3^	14.3 ± 33.5	11.1 ± 19.7	0.531
Brainstem volume, cm^3^	5.3 ± 0.8	4.4 ± 0.8	<0.001
Cerebellar volume, cm^3^	19.6 ± 5.9	13.8 ± 5.8	<0.001
Cerebrum volume, cm^3^	346.4 ± 61.8	292.5 ± 52.5	<0.001

Values are expressed as mean ± SD. Abbreviations: HCS, hydrocortisone.

**Table A3:** Predictive performance of cumulative hydrocortisone dose cutoffs for cerebral palsy.

Cumulative HCS Dose	>40 mg/kg	>57.5 mg/kg	>70 mg/kg	>90 mg/kg	>100 mg/kg	>120 mg/kg
Cerebral palsy	16/59 (27.1%)	15/50 (30.0%)	15/52 (28.8%)	14/39 (35.9%)	13/36 (36.1%)	12/34 (35.3%)
TP	16	15	15	14	13	12
FN	3	4	4	5	6	7
FP	43	35	37	25	23	22
TN	32	40	38	50	52	53
Sensitivity	84.2% (16/19)	78.9% (15/19)	78.9%(15/19)	73.7% (14/19)	68.4% (13/19)	63.2% (12/19)
Specificity	42.7% (32/75)	53.3% (40/75)	50.7% (38/75)	66.7% (50/75)	69.3% (52/75)	70.7% (53/75)
PPV	27.1% (16/59)	30.0% (15/50)	28.8% (15/52)	35.9% (14/39)	36.1% (13/36)	35.3% (12/34)
NPV	91.4% (32/35)	90.9% (40/44)	90.4% (38/42)	90.9% (50/55)	89.7% (52/58)	88.3% (53/60)
Accuracy	51.0%	58.5%	56.4%	68.1%	69.1%	69.1%
Youden Index	0.269	0.323	0.296	0.404	0.378	0.338
AUC (95% CI)	0.634 (0.533–0.736)	0.661 (0.551–0.771)	0.648 (0.538–0.758)	0.702 (0.587–0.817)	0.689 (0.569–0.808)	0.669 (0.546–0.792)

True positive, positive result on predictive test (High HCS) on presence of CP; False negative, negative result on predictive test (low HCS) on presence of CP; False positive, positive result on predictive test (High HCS) on absence of CP; True negative, negative result on predictive test (low HCS) on absence of CP. Accuracy = (TP + TN)/(TP + TN + FP + FN); Youden Index (J) = Sensitivity + Specificity−1. Abbreviations: AUC, area under curve; CI confidence interval; FN, false negative; FP, false positive; HCS, hydrocortisone; NPV, Negative Predictive Value; PPV, Positive Predictive Value, TN, true negative; TP, true positive.

**Table A4:** Logistic regression analysis model for the relationship between neonatal risk factors and long term outcome.

	CP			NDI			Head Circumference < 10th		
	OR	95% CI	*p* Value	OR	95% CI	*p* Value	OR	95% CI	*p* Value
Maternal characteristics									
Maternal age, years	1.10	0.97–1.24	0.124	1.02	0.93–1.11	0.713	0.99	0.88–1.12	0.879
Maternal hypertension	NA *	NA	0.989	0.72	0.22–2.31	0.580	0.99	0.22–4.54	0.991
Delivered by C/S	0.92	0.23–3.68	0.902	1.05	0.40–2.72	0.919	0.54	0.15–1.90	0.337
Histologic chorioamnionitis	1.04	0.36–2.95	0.946	1.38	0.64–2.99	0.414	0.69	0.24–2.00	0.493
Antenatal steroid	0.93	0.31–2.77	0.894	1.22	0.53–2.85	0.640	1.38	0.41–4.65	0.604
Infantile characteristics									
GA, weeks	0.98	0.77–1.26	0.899	0.9	0.75–1.06	0.210	0.88	0.70–1.12	0.304
BW, grams	1.00	1.00–1.00	0.942	1.00	1.00–1.00	0.259	1.00	0.99–1.00	0.009
5 min Apgar score	0.90	0.68–1.18	0.437	0.80	0.64–1.00	0.057	1.04	0.79–1.37	0.778
Male sex	2.48	0.85–7.21	0.096	1.74	0.84–3.64	0.138	1.57	0.59–4.19	0.369
Small for gestational age	0.41	0.05–3.43	0.409	0.72	0.22–2.31	0.580	3.17	0.69–14.6	0.138
Short-term Neonatal Outcomes									
Respiratory distress syndrome	NA *	NA	NA	NA *	NA	NA	0.60	0.04–9.94	0.718
Treated patent ductus arteriosus	2.92	1.04–8.20	0.042	2.17	1.03–4.68	0.045	2.58	0.93–7.15	0.067
Moderate to severe BPD	2.31	0.48–11.03	0.296	3.00	1.15–8.49	0.029	3.18	0.63–16.03	0.162
Necrotizing enterocolitis	NA *	NA	NA	0.68	0.16–2.69	0.578	0.81	0.14–4.78	0.818
Periventricular leukomalacia	6.75	2.17–21.00	<0.001	3.47	1.58–8.02	0.003	1.78	0.65–4.83	0.261
Intraventricular hemorrhage ≥ 2	1.33	0.34–5.18	0.678	2.92	1.21–7.48	0.020	1.44	0.44–4.75	0.546
Retinopathy of prematurity	0.99	0.19–5.07	0.986	1.02	0.32–3.37	0.972	0.43	0.08–2.24	0.315
Culture-proven sepsis	0.98	0.35–2.71	0.965	1.30	0.62–2.72	0.491	1.60	0.59–4.32	0.354
Duration of PN, days	1.00	0.99–1.02	0.452	1.00	0.99–1.01	0.471	0.99	0.98–1.00	0.188
Length of stay, days	1.01	1.00–1.02	0.09	1.02	1.01–1.04	<0.001	1.02	1.00–1.04	0.011
Regional Brain volume									
Intracranial volume	1.00	1.00–1.00	0.927	1.00	1.00–1.00	0.755	0.99	0.99–1.00	0.027
Ventricle volume	1.01	0.97–1.05	0.762	1.02	1.00–1.05	0.145	0.97	0.91–1.03	0.333
Brainstem volume	0.85	0.59–1.23	0.393	0.96	0.76–1.20	0.700	0.62	0.38–1.01	0.054
Cerebellar volume	1.00	0.97–1.04	0.946	0.99	0.97–1.02	0.642	0.93	0.88–1.00	0.039
Cerebrum volume	1.00	1.00–1.00	0.915	1.00	1.00–1.00	0.774	0.99	0.99–1.00	0.027
HCS > 90 mg/kg	5.60	1.81–17.31	0.003	3.79	1.74–8.61	0.001	7.42	2.49–22.15	<0.001

* Due to no events, in this group OR could not be estimated. Abbreviations: BW, birth weight; BPD, bronchopulmonary dysplasia; CP, cerebral palsy; C/S, Cesarean section; GA, gestational age; HCS, hydrocortisone; NDI, neurodevelopmental impairment; OR, odds ratio; PN, parenteral nutrition.
